# Safeguarding youth from agricultural injury and illness: The United States' experience

**DOI:** 10.3389/fpubh.2023.1048576

**Published:** 2023-01-30

**Authors:** Barbara C. Lee, Marsha A. Salzwedel

**Affiliations:** National Children's Center for Rural and Agricultural Health and Safety, Marshfield Clinic Research Institute, Marshfield, WI, United States

**Keywords:** children, USA, injuries, deaths, agriculture, safety, policy

## Background

The United States of America (US) is a country of 50 states with a population of more than 335 million people in 2022 (1). Across the expansive geography, there are ~2.1 million farms (2), with about 890,000 youth younger than 20 years old living and working on family farms, plus another 265,000 non-resident youth hired to work on farms each year (3). The tremendous diversity in US farm locations, size, commodities, machinery, and livestock is associated with a wide range of work assignments and risk exposures for young people of all ages. Occupational fatality data from the last decade indicate that, across all industries, agriculture had the leading number of work-related deaths for youth. Further, within the agricultural industry, youth between the ages of 10 and 15 suffered the most non-fatal work-related injuries (4). Although there are no official agricultural injury statistics for youth in the US, a 2014 government analysis estimated an annual 12,000 non-fatal injuries among youth and, of these, about 2/3 involved non-working youth, that is, individuals in the farm environment, but not actively engaged in the work itself (5). Leading causes of fatal and non-fatal injuries for both working and non-working youth are vehicles (including tractors, all-terrain vehicles, skid steer loaders), machinery, and contact with animals.^1^

Agricultural environments also include health risks that are compounded for youth in relation to their physical development stage. Concerns include exposures such as organic dusts, airborne pollutants, pesticides, toxic gases, and cleaning agents. Additionally, there are risks associated with heat-related illness, animal-transmitted infections, noise-induced hearing loss, musculoskeletal strains, sun exposure, and mental health (6).

The US child labor laws and policies are less restrictive than many international standards and are less restrictive for agriculture compared to other occupations. For example, the Fair Labor Standards Act (FLSA) allows youth starting at 12 years to be employed for farm work with unlimited hours, providing they have parental permission and continue to attend school. Family farms account for 96% of all US farms, and parents and guardians are exempt from compliance with the FLSA rules when their children are working on their own farms. The Child Labor in Agriculture Rules (https://www.dol.gov/sites/dolgov/files/WHD/legacy/files/childlabor102.pdf), including a list of Hazardous Occupation Orders restricting certain activities until age 16, have existed for nearly 40 years, and efforts to update them in 2011 were ceased primarily due to strong backlash from groups within the farming community (7, 8). Another difference in the US compared to many industrialized countries is the limited government support for working parents. Further, the US offers no universal paid parental leave, while childcare access in rural areas is both sparse and expensive (9, 10). These factors likely contribute to the increased presence of young children in agricultural work settings.

## Current activities

Since 1996, the US government, under leadership of the National Institute for Occupational Safety and Health (NIOSH), has supported research and interventions to address the complex factors contributing to preventable diseases and injuries affecting working and non-working children *via* a National Action Plan (11). Funds are allocated for independent research as well as support for a national coordinating center—the National Children's Center for Rural and Agricultural Health and Safety (NCCRAHS).

Many US organizations are promoting childhood agricultural safety and health, with NCCRAHS recognized as the national leader (12). Partnerships and collaborations are key to carrying out this work (12). Beginning in the 1990s, an important first step for interventions was the creation of voluntary guidelines for assigning youth work and building safe play areas for children on farms. Guidelines for agritourism (groups of children visiting on farms), media relations, and off-farm childcare followed in later years. Guidelines were drafted, refined and subsequently updated *via* consensus-development processes and/or advisory committees. Several were evaluated for their effectiveness and implementation strategies (13). All are available online, along with safety checklists, brochures, and public service campaign materials.

While NCCRAHS primarily targets strategies with farm parents, supervisors, and organizations that work directly with producers, other organizations such as Progressive Agriculture Foundation™ offer safety programs directly for children, with messages and demonstrations to convey principles of safety. “Safety Day” evaluations have shown their effectiveness (https://www.progressiveag.org/Success.cgi). Other relevant groups include AgriSafe and Ag Safety & Health Alliance that deliver programs for high school youth and college students.

The US Department of Agriculture (USDA) allocates funds for a national Safety for Agricultural Youth (SAY) online clearinghouse of work-related curricula, developed through a collaboration between Penn State, the Ohio State, University of Utah and Purdue University (ag-safety.extension.org/say-national-clearinghouse/). USDA also supports youth tractor certification courses delivered by state land grant institutions and county-level Extension services.

From 1998 to 2014 the US government systematically collected and reported childhood agricultural injury data providing empirical evidence on demographics, injury agents and trends (3). When that system ceased, AgInjuryNews.org was developed as a free, online, searchable database of publicly available news reports of youth and adult agriculture-related injuries; and is now expanding to include international data (14).

Beyond resources and injury data, many NIOSH-funded centers allocate funds for external projects through small grants programs for community-based organizations and/or junior faculty to build capacity in the discipline. In-person and virtual safety workshops and training events are collaborative efforts of NCCRAHS, Progressive Ag Foundation™, AgriSafe, the 11 regional NIOSH-funded agricultural centers, and several international partners. Nearly all organizations promote farm safety and health *via* social media, press releases, interviews, and conference presentations.

In 2009, the Childhood Agricultural Safety Network (CASN) (https://cultivatesafety.org/casn/) was established as a loose-knit coalition of child safety advocates, farm safety professionals, youth-serving organizations, health care providers, insurance agencies, and farm media. Coordinated by NCCRAHS, network members collaborate on education and outreach projects, including developing and disseminating public service campaigns on topics like “no extra riders on tractors” and ATV/UTV safety. Last year, CASN launched an online community to facilitate communication and collaborations. CASN has grown from an initial three to nearly 200 organizations in 2022, including 10 international members.

## Impacts and challenges

Over these past 25 years, there have been many accomplishments. Data showed a 60% decline in non-fatal childhood agricultural injuries from 2001 to 2014 (3). A national report placed the Childhood Agricultural Injury Prevention Initiative among the occupational safety “top 10” public health successes of the decade (15). Research confirmed the impact of work guidelines, demonstrating a 50% reduction in injuries where children's tasks were assigned per the guidelines (16). Funding has grown beyond NIOSH and USDA to include substantial private sector and agribusiness sponsorship of education, conferences and outreach efforts (17). Most importantly, positive impacts are attributed to the coordination and collaboration of many organizations raising the profile of childhood agricultural safety and health *via* education, training, interventions, and accessible resources.

Yet for all these accomplishments, challenges and gaps still persist. Myriad factors contribute to decisions regarding youth work on farms as well as presence of small children in the work setting. Farm parents often weigh the benefits of living and working on a farm with the risks of disease and injury in a concept known as the “Farm Kid Paradox” (18). Despite the evidence, studies reveal that some parents do not view the farm environment as dangerous and do not perceive their own children to be at risk of injury (19, 20). Another study revealed that parents often refer to “common sense” even when they are not modeling safe behaviors themselves (21). In contrast, some parents would prefer to keep children away from agricultural work areas, but have few alternatives because of the need to keep the farm operational amidst a lack of affordable and accessible childcare (22, 23). Adding to this is the challenge of reaching unique populations that harbor traditional practices and religious views regarding youth on farms, such as Plain communities (24). Furthermore, factors that compromise adults' safety behaviors have been exacerbated by the COVID-19 pandemic, their socio-economic status, and unstable labor markets (25).

A major challenge is the country's current child labor laws and policies that, if updated to be consistent with non-ag industries, could significantly reduce work-related child injuries and fatalities (26). Another concern is absence of timely, valid, and reliable injury data, especially since NIOSH's discontinuation of its Child Ag Injury Surveillance program (27). Also, the increasing use of small farm machinery, such as ATVs, UTVs, and skid steers has heightened risk exposures (28). Yet another challenge is the hesitancy of local officials, including Child Protective Services and District Attorneys, to hold adults accountable for endangering children in farm settings. On average only two childhood agricultural fatality or traumatic injury cases per year (of an estimated 100 deaths and 12,000 injuries/year) result in an adult being legally charged with a penalty for endangering or neglecting a child in a dangerous environment (29). To resolve these gaps and challenges we will need major financial investments, public policy, and “buy-in” from influential farm organizations.

## Future directions

Future attention must account for the ever-changing work practices, technologies, and risks of production agriculture, while respecting safe traditions, cultural beliefs, and social norms. To this end, the Socio-Ecologic Model (SEM) is an ideal framework for implementing youth agricultural safety and health strategies. The process encourages repeated approaches involving agents of influence at multiple levels—from individuals (e.g., parents), through community members (e.g., schools, churches), up to policymakers (30). This model incorporates perspectives from multiple angles toward a common goal.

National-level US approaches are dependent on government leadership with bipartisan support and involvement of agricultural stakeholders, but it is uncertain if that level of commitment will materialize, given other pressing priorities. The US' outdated child labor regulations, limited childcare services and labor shortages will continue to impact children's exposures to hazards on farms. Further, filling the childhood agricultural injury data gaps will require substantial funding and coordination across the many geographic regions. In addition to data, research is needed to better understand the barriers and motivators that influence parents' and supervisors' decisions to implement youth safety practices such as use of work or play guidelines.

Across the many US organizations, our goal continues to be the enhancement of health, safety, and wellbeing of all children living on, working on, and visiting US farms and ranches. We will continue to work collaboratively until every child is safeguarded from preventable disease and injury associated with agricultural environments.

## Author contributions

All authors listed have made a substantial, direct, and intellectual contribution to the work and approved it for publication.

## Footnotes

^1^In the US, the terms child, children and/or youth typically refer to any individual or groups younger than 18 years old. Some data sources refer to specific age groups and are such reported here. However, when the general term “youth” or “children” is used, it is interchangeable and implies individuals 0 through 17 years.
